# COVID-19 crisis, safe reopening of simulation centres and the new normal: food for thought

**DOI:** 10.1186/s41077-020-00131-3

**Published:** 2020-07-16

**Authors:** Pier Luigi Ingrassia, Giorgio Capogna, Cristina Diaz-Navarro, Demian Szyld, Stefania Tomola, Esther Leon-Castelao

**Affiliations:** 1 Centro Interdipartimentale di Didattica Innovativa e di Simulazione in Medicina e Professioni Sanitarie, SIMNOVA, Università del Piemonte Orientale, Via Lanino 1, 28100 Novara, Italy; 2 Scuola di Anestesia, Centro di Simulazione EESOA, Rome, Italy; 3 Department of Peri-operative Care, Cardiff and Vale University Health Board, Cardiff, UK; 4 Center for Medical Simulation, Department of Emergency Medicine, Brigham and Women’s Hospital and Harvard Medical School, Boston, MA USA; 5 Centro di Simulazione (CeSi) at the Centro Professionale Sociosanitario, Lugano, Switzerland; 6 Clinical Simulation Laboratory, School of Medicine and Healthcare Sciences, University of Barcelona, Barcelona, Spain

**Keywords:** Simulation, Simulation centre, COVID-19, SARS-CoV-2, Coronavirus, Lockdown, Training

## Abstract

**Background:**

The world is facing a massive burden from the coronavirus disease 2019 (COVID-19) pandemic. Governments took the extraordinary step of locking down their own countries to curb the spread of the coronavirus. After weeks of severe restrictions, countries have begun to relax their strict lockdown measures. However, reopening will not be back to normal.

Simulation facilities (SF) are training spaces that enable health professionals and students to learn skills and procedures in a safe and protected environment. Today’s clinicians and students have an expectation that simulation laboratories are part of lifelong healthcare education. There is great uncertainty about how COVID-19 will impact future training in SF. In particular, the delivery of training activities will benefit of adequate safety measures implemented for all individuals involved.

This paper discusses how to safely reopen SF in the post-lockdown phase.

**Main body:**

The paper outlines 10 focus points and provides operational tips and recommendations consistent with current international guidelines to reopen SF safely in the post-lockdown phase. Considering a variety of national advices and regulations which describe initial measures for the reopening of workplaces as well as international public health recommendations, we provide points of reflection that can guide decision-makers and SF leaders on how to develop local approaches to specific challenges. The tips have been laid out taking also into account two main factors: (a) the SF audience, mainly consisting of undergraduate and postgraduate healthcare professionals, who might face exposure to COVID-19 infection, and (b) for many simulation-based activities, such as teamwork training, adequate physical distancing cannot be maintained.

**Conclusions:**

The planning of future activities will have to be based not only on safety but also on flexibility principles.

Sharing common methods consistent with national and international health guidelines, while taking into account the specific characteristics of the different contexts and centres, will ultimately foster dissemination of good practices.

This article seeks to further the conversation. It is our hope that this manuscript will prompt research about the impact of such mitigation procedures and measures in different countries.

## Background

The world is facing a massive burden from the coronavirus disease (COVID-19) pandemic.

As the number of infections increased exponentially since the first case of COVID-19 was recorded, healthcare services in different countries faced increasing pressure to manage high volumes of critically ill patients, with some healthcare systems close to collapse [1]. On March 9th, the Italian government took the extraordinary step of locking down the country to curb the spread of the coronavirus. Quarantines, closure of schools, universities and all non-essential businesses, as well as social distancing, were all critical actions aiming to slow viral transmission, minimize surges in healthcare demand and buy time for researchers to try to develop therapies and a vaccine. Since then, similar countermeasures against the pandemic have been adopted in several European Union (EU) member states and non-EU countries with a full or partial lockdown in place [2].

Recently, countries, including Spain, Iran, Italy, Denmark, Israel, Germany and the USA, have cautiously announced and put in place plans to lift coronavirus lockdown measures over the coming days, seeking to pave the way for a return to a semblance of normal life after weeks of severe restrictions. However, reopening will not be back to normal. Nobody can now predict exactly when or if the virus will be defeated and what damage will remain, especially for the economy. The only certainty is that life after COVID-19 will be different.

Simulation-based education has been rapidly developing. Clinical situations for teaching and learning purposes can be created using mannequins, part-task trainers, simulated patients or computer-generated simulations. Simulation-based learning can be adapted to cater for the learners abilities, set up at appropriate times and locations and repeated as often as necessary and in a standardized manner. Simulation has not only been used to educate and train healthcare personnel but also to identify and test effective safety interventions to improve safety-oriented behaviour and subsequent patient outcomes [3]. However, the limitations of simulation, such as the need of dedicated and exclusive resource personals, technical and programming difficulties and the considerable costs, have to be recognized as well [4].

Simulation has been demonstrated to have a great potential to contribute to the management of not only previous pandemics [5–9] but also the current global COVID-19 crisis [10–13].

Simulation facilities (SF) are training spaces that enable health professionals and students to learn skills and procedures in a safe and protected environment. Today’s clinicians and students have an expectation that simulation laboratories are part of lifelong healthcare education [14].

There is great uncertainty about how COVID-19 will impact future reopening of SF. The evolution of the COVID-19 epidemic calls for the reconsideration of all activities performed in them. In particular, the delivery of training activities will benefit of adequate safety measures implemented for all individuals involved.

The aim of this paper is to outline 10 focus points and provide operational tips and recommendations consistent with current international guidelines to reopen SF safely in the post-lockdown phase. Considering a variety of national advice and regulations which describe initial measures for the reopening of workplaces as well as international public health recommendations, we provide points of reflection that can guide decision-makers and SF leaders on how to develop local approaches to specific challenges. The following tips have been laid out taking also into account two factors: (a) the SF audience, mainly formed by undergraduate and postgraduate healthcare professionals, who might face exposure COVID-19 infection, and (b) for many simulation-based activities, such as teamwork training, adequate physical distancing cannot be maintained.

This article seeks to further the conversation. Undoubtedly, varying opinions will be published as the field continues to develop.

## Main text

### Focus point 1: Establish a COVID-19 response task force

With so much discussion on how to safely reopen the workplace, SF leaders might be encouraged to think holistically about the choices they must make. Establishing a COVID-19 task force can help to develop the creative and pragmatic solutions that this exceptional time requires. This task force should be formed with the mission to ensure a risk assessment is performed and a thorough and intentional action plan is developed in order to support the SF activities from a safety, operational and also business risk perspective. The task force might include, at a minimum, members of SF management and representatives from the following functional areas: human resources, faculty, simulation and information technology and office administration or facilities. The incorporation of infection control practitioners should also be considered. Ideally, all issues related to your COVID-19 response would be led by, or funnelled through, this task force. Essentially, the COVID-19 task force should constantly assess the situation as it evolves, monitor the results of actions taken and adjust them if required.

### Focus point 2: Use of space

Use of SF space will need to be reviewed and adapted in order to provide safe environments. Until now, infection control has not been a focus in SF design with regard to either physical distribution or policy writing [15]. Simulation facility leaders should review current space use and plan new designs. Reducing crowding and maintaining social distance have been key policy measures to reduce the transmission of COVID-19 [16]. Thus, space planning should include those measures. A tentative classification of the areas of the SF is shown in Table 1.

**Table 1:** Classification of SF areas based on crowding

Class	Denomination	Description	Example	Recommendations
A	Walk-through areas	Areas where people will pass through without stopping	Hallway, lobby, parking lot	Consider creating one-way routes
B	Short stay areas	Areas where people can only stop briefly, 15 min at the most	Hall, toilet room	Consider placing physical distancing footprint floor signs in waiting areas
C	Prolonged stay areas where adequate physical distancing can be maintained	Areas where people are expected to stay for longer than 15 min, even several hours	Offices, classrooms, debriefing rooms, control rooms	It is mandatory to wear surgical face masks and to observe physical distancing. Shoe covers might be considered. These areas should also be adequately ventilated.
D	Prolonged stay areas where adequate physical distancing *cannot* be maintained	Areas where people are expected to stay for longer than 15 min, even several hours	Advanced simulation rooms, laboratories with task trainers	It is essential to wear other protective devices (e.g. gloves, goggles or visors, waterproof gowns) besides surgical face masks. All environmental surfaces should be cleaned and sanitized at the end of each simulation scenario. Rooms should also be adequately ventilated
E	Gathering areas	Indoor or outdoor areas where large groups of people are expected to gather in limited spaces	Main entrance, reception desk, toilet room entrance elevator, stamping area	It is essential to avoid gatherings whenever possible. Potential strategies include staggered entries, staff shift review, etc.
F	Gathering areas where the use of PPE is not mandatory	Areas where people are expected to stay to eat and drink, not wearing face masks	Lounge area, canteen	Packed lunches could be provided or attendees could be encouraged to bring their own lunch to be consumed outdoors or in the classroom.

In general, where technically possible, air recirculation systems should be avoided and adequate air exchange and natural or forced ventilation of the rooms should be ensured instead [17].

#### Layout and route review

The flow of movement within simulation premises will need to be considered. In particular, the points of entry to the facility should be different from the exit points if possible, so that people inside follow a one-way route and avoid crossing paths. The number of people authorized to take each elevator simultaneously should also be reduced (i.e. by limiting the number of people allowed in the elevator according to half its maximum weight capacity) and, wherever possible, the use of stairs should be encouraged instead.

Floors should be marked to aid physical distancing. Facility managers should consider appropriate marking for offices, classrooms, debriefing areas, control and simulation rooms (Fig. 1): from squash-court-style lines in lobbies and waiting areas to footprints signalling standing spots in lifts, circles around desks and lanes in corridors [18]. Walls could also display visual instructions. Examples of printable stickers are provided in Fig. 2.

**Fig. 3:**
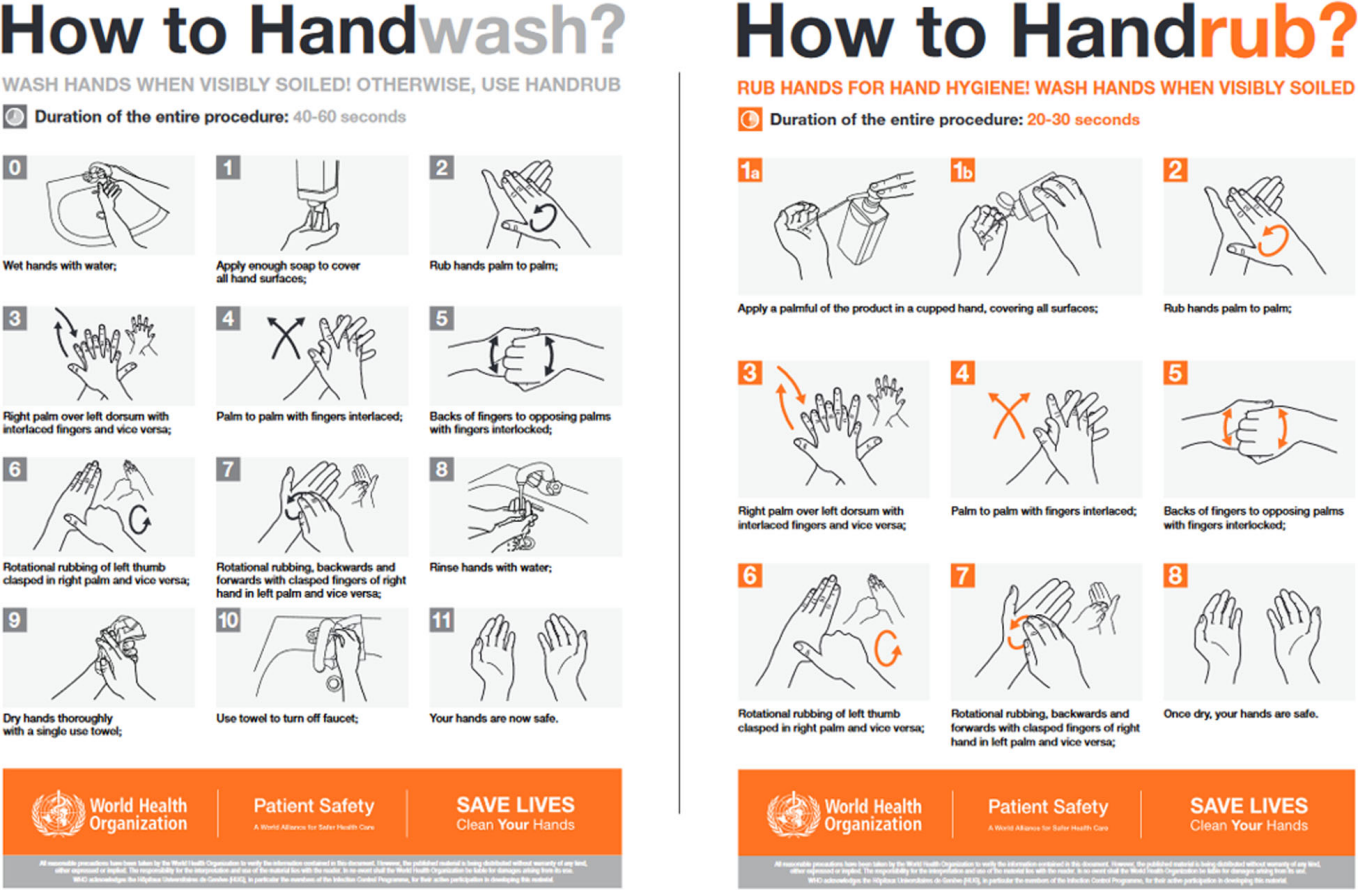
WHO posters on how to rub and wash hands. (Source: WHO Guidelines on Hand Hygiene in Health Care. World Health Organization 2009)

**Fig. 1:**
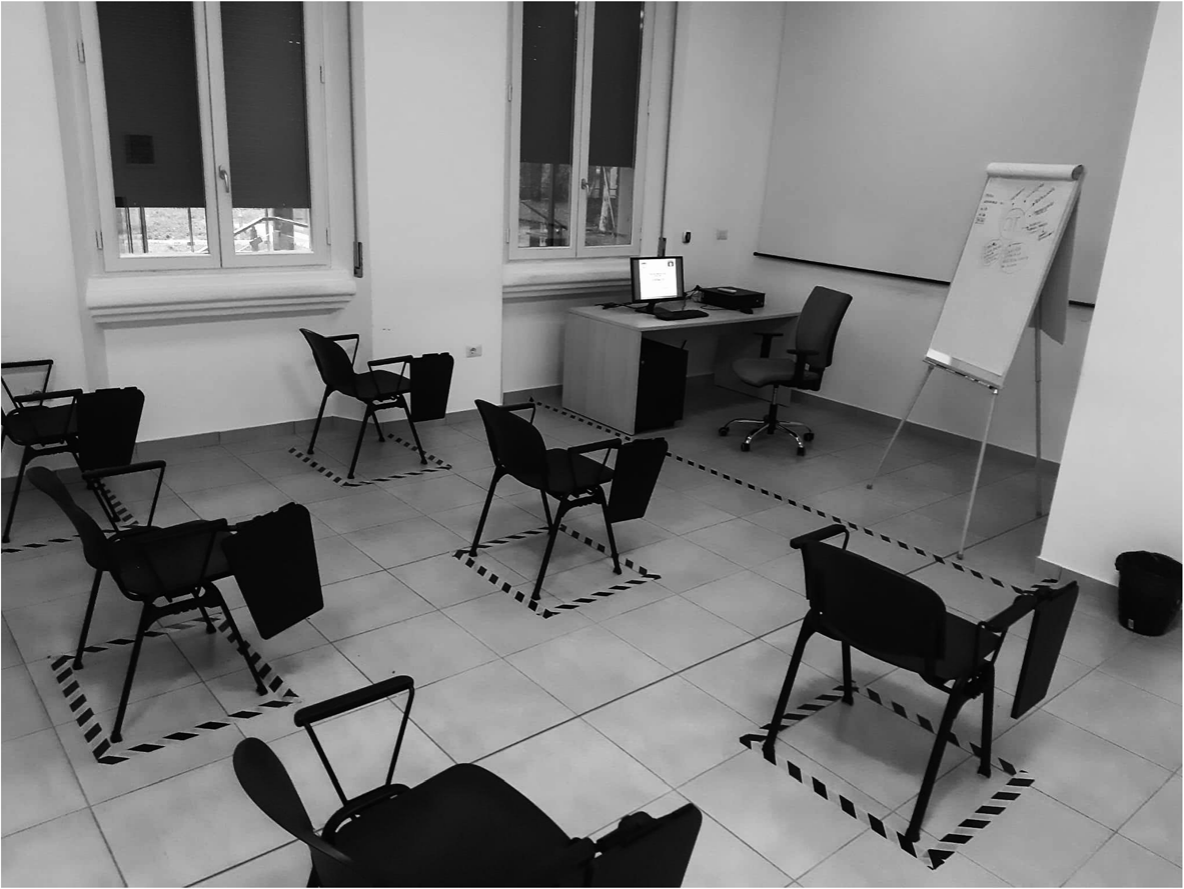
Physical distancing floor signs in a lecture hall

#### Management of common areas

Access to common areas, such as canteens, smoking areas and dressing rooms and drink and snack vending machines, should be strictly regulated in compliance with national recommendations. Measures could be put in place to reduce the transit time in these areas and ensure that people maintain a safe physical distance of at least 1 m apart [19]. Furthermore, people should avoid facing each other when distancing is limited. In rest rooms, hand dryers might need replacing with disposable wipes to avoid dispersion of droplets [20].

### Focus point 3: Conditions of access to the simulation facility

#### Communicate with students, faculty and staff in advance

The SF should promptly and effectively inform staff, faculty, students and visitors of the COVID-19 guidelines issued by local and/or national authorities. This can be achieved by e-mailing or posting them in advance and displaying them at the entrance or other prominent places at the premises. Information provided could include a summary of the applicable guidance along with details on personal data processing.

Access to the SF could be conditional to agreement to the following points:

- Attendance should be refused if an individual exhibits a fever (37.5 °C or more) [21] or other flu-like symptoms. In that case, they would be advised to consider contacting their family doctor and follow contemporaneous public health guidance.
- All attendees must promptly and responsibly inform staff members of the appearance of any flu-like symptoms during their attendance to training activities, while making sure to stay at a safe distance from other people.
- Staff members and students should ensure that they have fully recovered from any previous COVID-19 infection. This might include submission of a medical certificate.
- All attendees must comply with local and national public health recommendations. In particular, maintaining a safe distance from others, observing the rules of hand hygiene and other appropriate hygiene behaviours.

#### Health and safety checks

Before entering the SF, all faculty, staff and students might be asked to complete a health declaration form and undergo temperature checks. Additional health information may also be requested, in compliance with confidentiality requirements and current privacy regulations. An example of health declaration form for those entering the centre is provided in Additional File 1.

Safety checks should be performed by trained personnel wearing the appropriate personal protective equipment (PPE). If an individual’s core temperature is higher than 37.5 ° C, access to the premises should be denied. All symptomatic individuals should be immediately provided with a surgical face mask if they are not already wearing one and should be advised to follow contemporaneous guidance on self-isolation, always ensuring the confidentiality and dignity of the subjects. They also could be advised to contact their family doctor or the designated local health authority if appropriate.

Before entering the facility, staff and students should wash their hands or decontaminate them with alcohol-based hand rub, wear surgical face masks and follow, for the entire duration of the training activities, including breaks, the physical distancing rules explained by the staff and indicated on the posters and other signage, which should be clearly visible.

Shoe covers are not specifically recommended in the current guidance on PPE in the context of COVID-19 [22]. Nonetheless, considering the evidence that the soles of medical staff shoes might function as virus carriers [23], SF might consider the use of disinfectant rubber-based mats at the entrance especially for those facilities located within healthcare premises.

#### Contact tracing

Contact tracing is a key strategy for preventing further spread of COVID-19 and is included in multipronged plans to fight the pandemic [24]. Therefore, there should be a record of all individuals attending each educational activity and their contact details, to be used in case that any simulation participants develop COVID-19 symptoms. Processing of this information must be carried out in strict compliance with national general data protection regulations.

#### Care and storage of personal items

The coronavirus that causes COVID-19 is relatively new, and experts are still learning about its transmissibility from contaminated inanimate surfaces, such as fabric [25]. In this uncertainty, jackets, coats and other garments should not be placed on chairs and sofas. Clothes should be stored in dedicated areas or sealed in plastic bags instead. Likewise, all participants, especially those who have recently travelled, should store their suitcases and bags in dedicated areas accessible by separate routes.

#### Suppliers and visitors

Supplier access should be tightly regulated by identifying and implementing suitable entry, transit and exit procedures and by adopting appropriate delivery methods, routes and schedules. Drivers should remain inside their vehicles whenever possible. During loading and unloading of supplies, individuals must maintain a physical distance of at least one meter (3.3 ft) [26].

Access by visitors should also be restricted to necessary cases only. If the presence of external visitors is essential (e.g., guests, cleaning or maintenance company workers), they should comply with all the recommendations previously described.

### Focus point 4: Personal hygiene precautions and protective equipment

All people present in the facility should take all recommended hygienic precautions, especially those regarding hand washing [27]. SF must provide suitable hand cleansing agents. For hand washing, ordinary soap is sufficient. In the absence of water, alcohol-based sanitizers could be used as recommended by the World Health Organization (WHO) [28]. Hand rub formulations can also be prepared by SF according to WHO guidelines [29]. Instructions on how to rub and wash hands should be provided and WHO published posters could be used (Fig. 3).

**Fig. 2:**
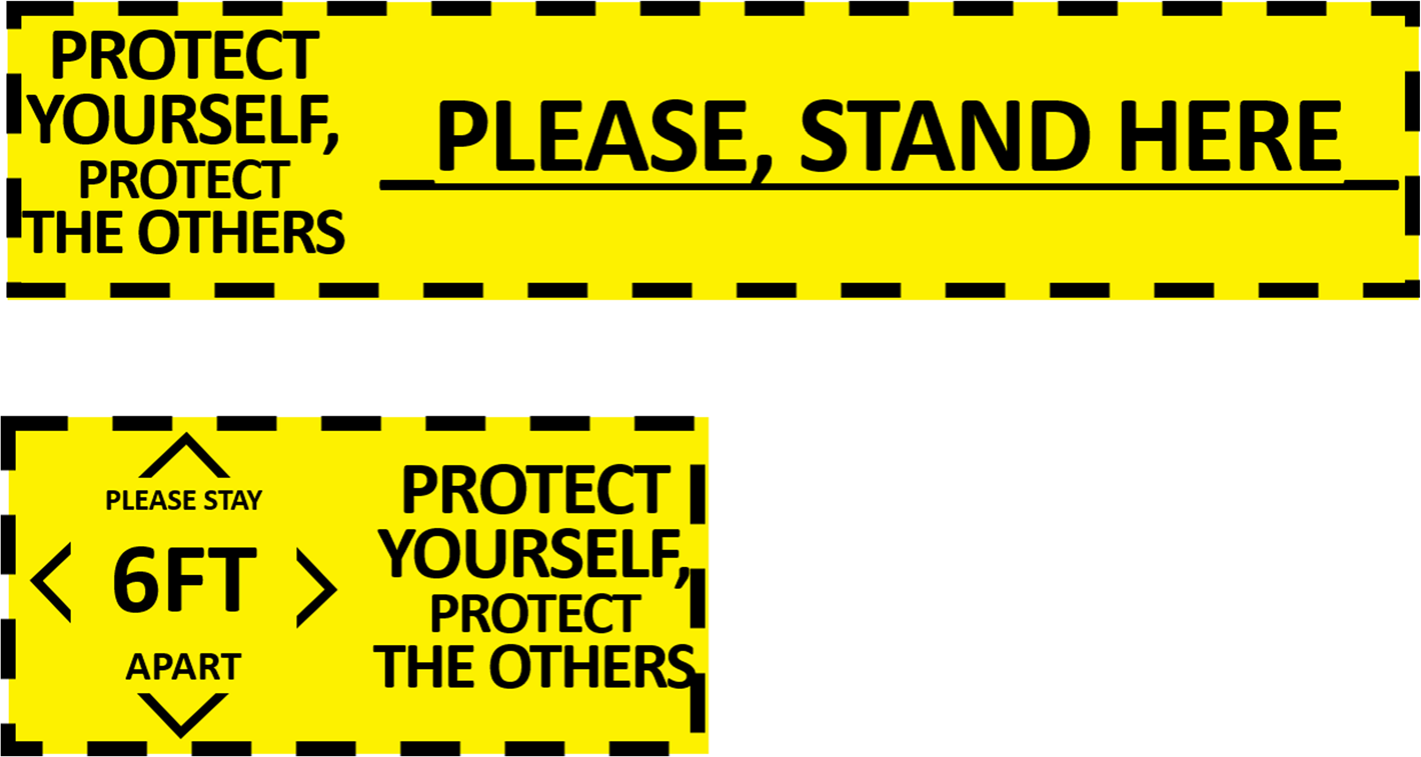
Examples of printable social distancing floor signs

Staff, facilitators and students should be asked to wear masks for the entire duration of all educational and administrative activities [30–33]. A recent meta-analysis of randomized controlled trials showed that surgical masks are as effective as N95 masks in reducing transmission of influenza-like diseases [34].

During participation in simulation scenarios, when not appropriate to maintain a physical distance greater than 1 m, involved participants should wear, besides face masks, other protective equipment (e.g. gloves, goggles or visors and waterproof gowns).

Doffing after the scenario should take place in a predetermined ventilated area with adequate distance between individuals. Lockers should be made available to students and staff if possible, to allow for appropriate storage of clothing and personal belongings.

When training activities last several hours, faculty and learners should be encouraged to bring their own food where possible. Personal cutlery and drinking cups should be advised.

### Focus point 5: Management of physical distancing during training activities

Social distancing is the most effective preventative strategy since the emergence of COVID-19 pending development of a vaccine, treatment or both [34]. Therefore, educators need to consider reorganizing their training sessions. For instance, they could consider delivering more editions of a given training activity but with a lower number of participants, thereby ensuring that physical distancing norms can be followed.

Creative and innovative approaches to simulation activity design might be welcome by students and staff. Although this topic would be amenable to a separate discussion, we suggest that elements to explore could include online sessions, virtual reality, outdoor debriefings, etc.

**Fig. 4:**
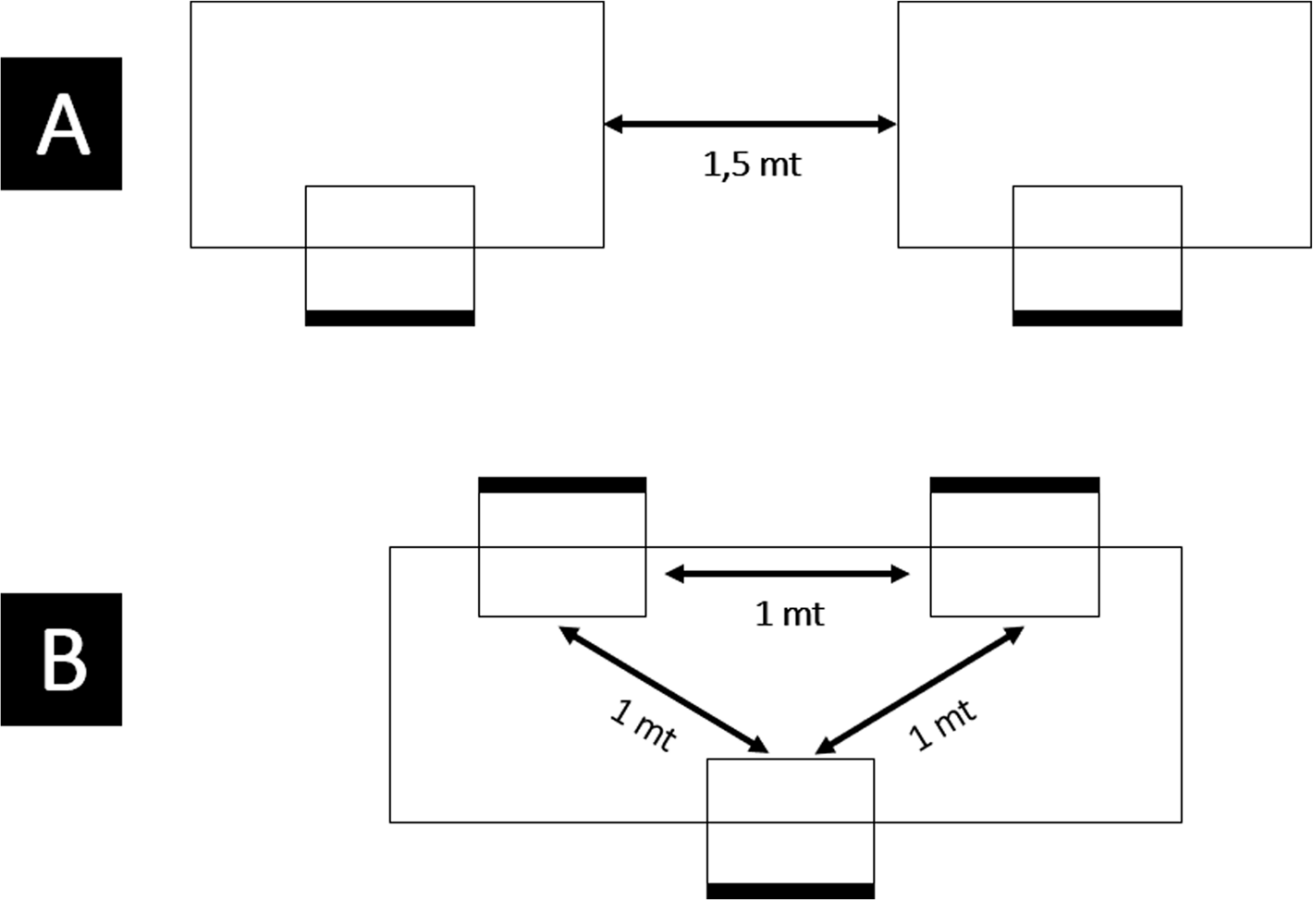
Example of checkerboard type seating pattern

When classroom activities are deemed necessary, it is recommended to stagger the arrival and to reduce the number of students to allow appropriate physical distancing. In rooms equipped with fixed seats and/or tables and in light of the required reduction of close contacts between the students, even if they are wearing face masks, a distance of at least two seats must be guaranteed at all times. In rooms where seats and tables can be freely moved, it is advisable to at least half the capacity of the classroom according to guidelines for public places. In any case, tables must be at least 1.5 m apart (Fig. 4a) and people must be seated according to a checkerboard seating pattern (Fig. 4b). If this measure does not provide a minimum separation of 1 meter, it will be necessary to further reduce the maximum capacity of the room. Student access might also be limited through staggering attendance in order to avoid gatherings.

**Fig. 5:**
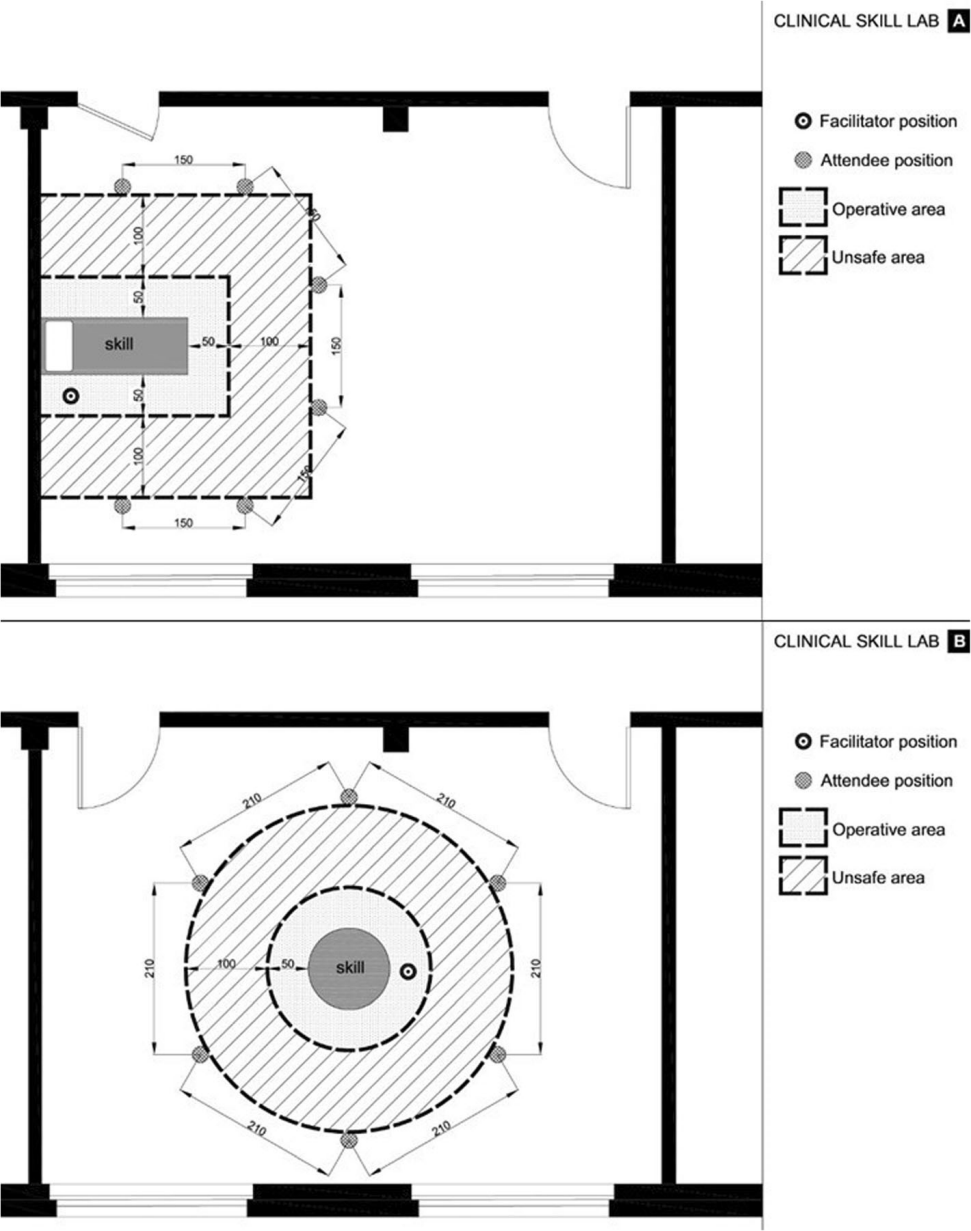
Example of clinical skill simulation lab layout

Simulation skill rooms might need reconfiguring and probably reduce their capacity to keep enough space between participants and faculty members (Fig. 5). Additionally, consideration should be given to ensuring maximum ventilation and minimizing air recirculation throughout [20].

### Focus point 6: Management of staff

Staggered entry/exit times should be planned in order to avoid physical proximity in common areas (e.g. entrances, reception desks, dressing rooms).

Temporary suspension of co-working arrangements that do not guarantee minimum safety distances between people according to national public health guidance should be considered unless workstations are separated by cardboard or plastic dividers. Use of these screens is also recommended to protect staff working at reception desks.

In compliance with WHO recommendations, SF should implement flexible working arrangements for all those activities that can be performed at home [26]. Effective communication and support from the SF managers and colleagues should be guaranteed. Having regular staff or team meetings held online should also be considered.

Online meetings would be preferable to face to face meetings. If meeting participants lack adequate remote connections and the meeting is urgent, the number of attending participants should be reduced to a minimum to ensure appropriate physical distancing. Adequate cleaning and ventilation of the premises where the meeting will be held must be ensured. In addition, it will be necessary to collect details of all participants to be able to inform them retrospectively in the event of any participant developing symptoms of COVID-19 during or after the meeting.

National and international business trips would not be currently recommended by most countries and organizations. Further travel should follow up to date national and international recommendations.

*Sickness tracking* and *absence* management has become of new critical importance. All COVID-19 sickness absences should be recorded within a special leave category as opposed to ‘normal’ sickness absence to help SF monitor the impact of the epidemic on the workforce and to report this impact to local public health authorities.

### Focus point 7: Cleaning and disinfection

The COVID-19 virus spreads mainly from person to person through respiratory droplets produced when an infected person coughs or sneezes. It may be possible for a person to get COVID-19 by touching a contaminated surface or object and then touching their own mouth, nose or eyes [35].

The SF should ensure regular cleaning and periodic sanitization of the premises including simulation environments, debriefing rooms, workstations and common areas. After each scenario it should be considered whether environmental surfaces, manikins and reusable materials should be disinfected (has hand hygiene been complied with? has the appropriate PPE been worn by all individuals present?). Recruiting a trained observer for monitoring doffing and hand hygiene post scenario could be helpful.

#### Cleaning

The term ‘Cleaning’ refers to the removal of germs, dirt and impurities from surfaces. It does not kill germs, but by removing them, it lowers their numbers and the risk of spreading infection. According to Centre for Disease Control and Prevention (CDC), routine cleaning of frequently touched surfaces (i.e. tables, doorknobs, light switches, handles, desks, toilets, faucets, sinks and electronics) can be conducted by household cleaners and disinfectants that are appropriate for a given surface, following the label instructions [36].

The cleaning plan should include:

- Administration and trainers’ offices
- Simulation rooms and control rooms, paying special attention to the most frequently touched surfaces such as trolleys, drawer handles, tables, electro-medical devices (e.g. monitor surfaces, defibrillator handles and pressure cuffs), tools and devices (e.g. laryngoscopes), doors, handles, taps, sinks, cell phones and keyboard
- Classrooms and debriefing rooms, with particular attention to the most frequently touched surfaces such as desks, chairs, armrests, keys, keyboards and printers
- Common areas
- Refreshment and canteen areas
- Toilets and dressing rooms
- Elevators, vending machines for drinks and snacks, with particular attention to the surfaces most frequently touched, such as doors, handles and elevator buttons
- Personal work tools. Daily cleaning of work tools should be carried out by each worker at the end of the shift. To this end, the worker should be provided with suitable cleaning agents and adequate instructions.

In all cases, it is recommended to wear gloves when cleaning and to avoid splashing and spraying around during these tasks.

#### Disinfection

The term ‘disinfection’ defines the act of decontaminating or reducing the viral load with specific chemical solutions. By killing germs on a given surface after cleaning, it can further lower the risk of spreading infection. With regard to the environments and surfaces (i.e. of monitor touch screens, keyboards, mouse devices), in accordance with the guidelines issued by the WHO, a thorough cleaning with microfiber cloths moistened with water and detergent followed by the application of disinfectants routinely used in hospital is deemed an effective and sufficient disinfection. The COVID-19 virus is indeed effectively inactivated by adequate sanitization procedures using disinfectants, such as sodium hypochlorite (NaClO) (0.1–0.5%), ethanol (62–71%) or hydrogen peroxide (0.5%), for an adequate length of time (Table 2) [38]. The United States Environmental Protection Agency listed all products for use against COVID-19 [39].

**Table 2:** Cleaners and disinfectants. (Source: Kampf et al. [37])

Disinfectant	Concentration	Effective Exposure
Ethanol	70%	10 min
Sodium hypochlorite	0.01%	10 min
Hydrogen peroxide	0.5%	1 min
2-Propanol (isopropyl alcohol)	70%	30 s
Bleach	0.21%	30 s

Following each disinfecting intervention, ventilation must always be ensured to avoid the risk of inhaling toxic fumes.

**Fig. 6:**
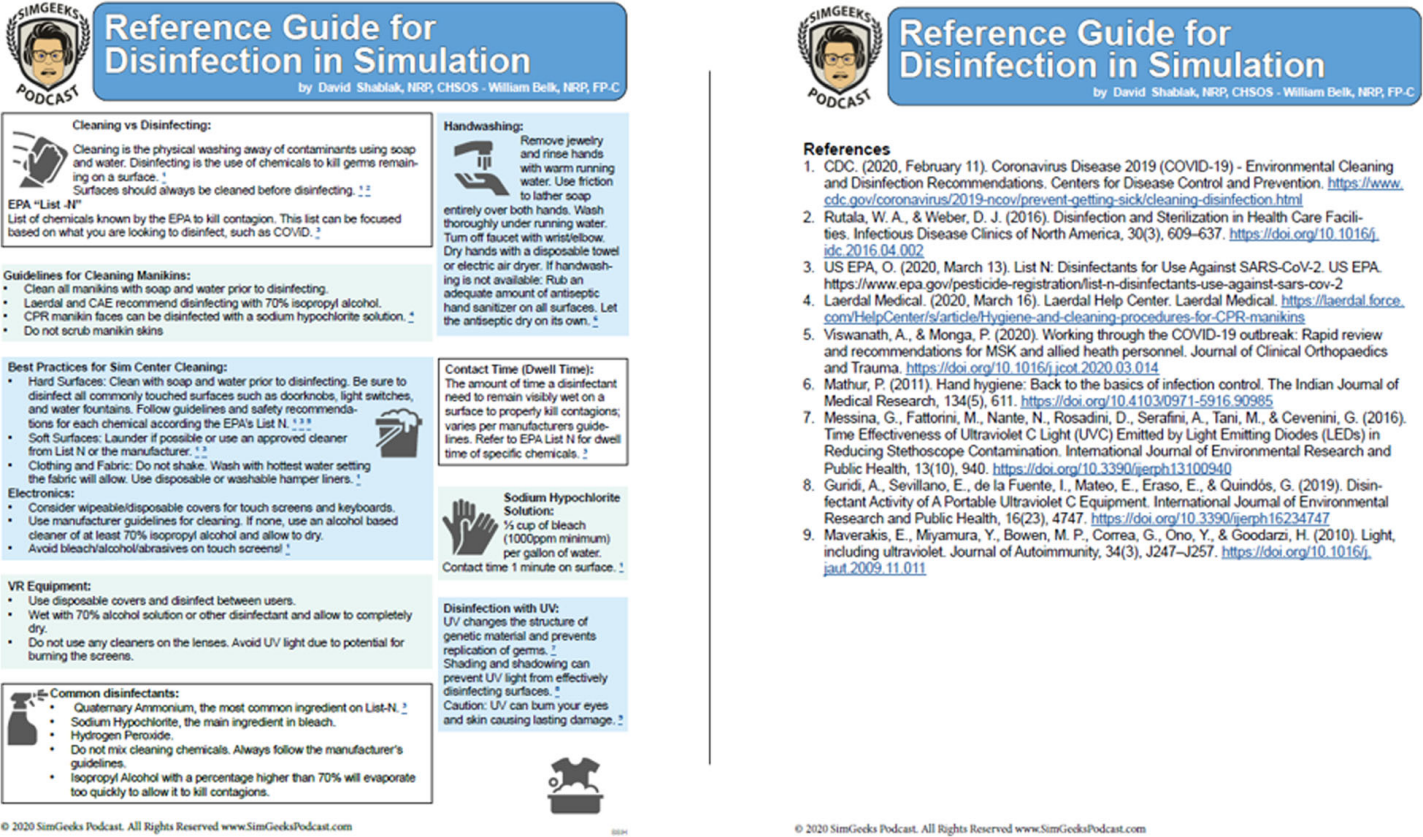
Reference guide for disinfecting in simulation (by permission of Shablak D. and Belk W.)

It is always recommended to follow manufacturer’s instructions for cleaning and disinfection of the mannequins and part-task trainers. Table 3 provides additional manufacturer’s information. Anyway, a reference guide for disinfecting in simulation is provided in Fig. 6.

**Table 3:** Instructions for cleaning and sanitization of simulators and task trainers

	Standard cleaning	Disinfection	Additional instructions
**Lifecast—Body Simulation**	Use a mild liquid soap solution.	Use chemical disinfectants—according to the manufacturer’s instruction, these chemicals are indicated for silicone surfaces. Rinse thoroughly with clean water and leave to dry before storing it away. Alcohol wipes (based on solutions > 60% alcohol) may also be used by gently rubbing the surface.	Mouth-to-mouth ventilation is not recommended. For more info visit: https://www.lifecastbodysim.com/
**Simulab Corporation**	Use a solution of mild liquid soap and warm water. Dry with a soft cloth.	Spray isopropyl alcohol on the simulator and clean it with a soft cloth. Alcohol is also effective in removing any stains.	Do not clean with chemical solvents. Do not use abrasive sponges. If the simulator includes electronic components, make sure that these are not exposed to any moisture. Make sure the simulator is completely dry before storing it away. For more info visit: https://www.simulab.com/covid-19-resource-center
**Gaumard**	Use a cloth moistened with diluted liquid detergent (dish soap).		Clean any traces of adhesive with alcohol wipes. Do not use solutions containing citric acid (it can cause corrosion). Do not immerse the simulator in water. For more info visit: https://www.gaumard.com/downloads
**Operative Experience**	Cleaning simulators can be wiped clean with a simple solution of mild soap and water. Insert the vent plugs into the ventilation openings to protect the electronics when washing the unit.		For more info visit: https://operativeexperience.com/support/
**3B Scientific**	Use a cloth moistened with a soap and water solution.	Use a cloth moistened with alcohol.	Do not use pure alcohol directly on the silicone. Do not use abrasive or corrosive detergents. For more info visit: https://www.3bscientific.it/
**Laerdal**	Use a cloth moistened with a soap and water solution. Facial skin and other rigid plastic parts can be disassembled and immersed in water at 60 – 70 °C containing dishwashing detergent for 20 minutes. Rinse and dry the components thoroughly	The facial mask and facial connector of the simulator can be cleaned with sodium hypochlorite solution (NaClO).	During a CPR session, thoroughly disinfect the face of the manikin after each use (student) using disinfectant wipes. The airways are disposable and should be replaced after a CPR lesson if mouth-to-mouth ventilation has been performed. For more info visit: https://laerdal.force.com/HelpCenter/s/article/Hygiene-and-cleaning-procedures-for-CPR-manikins
**CAE**	Use a mild detergent and warm water to remove most marks and stains. Gently rub the soiled area with a soft cloth. Do *not* use abrasive soaps or pads.		For more info visit: https://caehealthcare.com/covid19/
**Blue Phantom**	Cleaning your training model after each use, your training model can be easily cleaned using mild soapy water. For best results, mix one part liquid soap with one part tap water. Gently rinse the model with the soapy water to remove any accumulated debris. Use a clean, soft, lint-free cloth to dry after cleaning. Dry the model using a dabbing motion, rather than wiping or rubbing the model.		For more info visit: http://info.bluephantom.com/blue-phantom-user-guides

Disclaimer: What is recommended in the above table derives from indications sent directly by Italian companies or distributors. The authors decline any responsibility for the information provided on this table

#### Reusable materials

Maintaining efficient levels of reusable medical equipment is vital for SF due to their high cost [40]. In fact, during simulation-based training, learners are asked to perform tasks as they would do during real patient encounters. This includes using disposable medical supplies and equipment needed to perform a procedure or clinical skill, for instance syringes. These have been commonly reused in order to reduce costs. In view of new requirements to disinfect equipment, educators and SF managers need to consider the cost-effectiveness of various alternatives. Based on our experience, a reasonable option might be the use of chlorine/NaDCC tablets diluted in water for disinfection of reusable kit. The equipment can be either submerged in the solution or washed with it, then should be left to dry naturally. Afterwards, it should be washed again with soapy water.

Linen and other fabric materials, where deemed essential to the simulation activity, should be washed with appropriate detergent and at the hottest possible temperature according to the manufacturer’s instructions. Adding a washing cycle with bleach or NaClO-based products may be considered, particularly if washing at high temperatures is not recommended for the garment [41].

#### Cleaning and disinfection when there are suspected or confirmed cases of COVID-19

In accordance with European Agency for Safety and Health at Work (EU-OSHA) [42], if a person becomes sick during the day, all surfaces that the person has come into contact with should be cleaned, particularly all surfaces and objects which are visibly contaminated with body fluids, but also all potentially contaminated high-contact areas such as toilets, door handles, monitors and cart handles.

Areas where a COVID-19 suspected individual has passed through and spent minimal time in (such as corridors or halls) but which are not visibly contaminated with body fluids should not need to be specially cleaned and disinfected. Shared spaces, such as lecture halls or debriefing rooms, should be cleaned using disposable cloths and usual cleaning products, according to current recommended workplace legislation and international guidelines. Consider opening outside doors and windows to increase air circulation in the area. The US CDC recommends waiting 24 h or as long as practical before beginning cleaning and disinfection [43].

#### Management of waste derived from cleaning and disinfection

Waste derived from cleaning activities should be collected in bags, sealed and disposed of in the mixed waste bin.

Waste that has been in contact with an individual with suspected or confirmed COVID-19 or produced by disinfection after contact, i.e., cleaning cloths and disposable PPE, should be collected separately, double bagged and disposed of as hazardous material [42].

### Focus point 8: Test your new set-up

The application of simulation in healthcare extends into the realm of process and systems testing. In fact, conducting simulation provides a unique opportunity to identify system errors and latent hazards [3, 4, 7, 8]. SF leaders should consider testing the new mitigation procedures through simulation to evaluate personnel flow, possible system weaknesses and inefficiencies.

A process map, which details each sequence of events related to the processes being evaluated, is recommended. Detailed notes and standardized evaluation templates could help to identify any opportunities for improvement and any corrective actions that need to be taken.

### Focus point 9: Management challenges

Due to Covid-19, these are highly uncertain times with frequent changes to local and external regulations. To cope with these challenges, SF directors and executives will need to question, reassess and redefine their managerial thinking. Three key areas to address are human resources, materials and equipment and budget reallocation.

#### Human resources

When possible, SF directors and managers need to consider separating faculty and technicians into different teams to preserve healthy workforce. In case of COVID infecting a team member, its transmission should be contained to that group (i.e. imagine having your entire nursing faculty in quarantine during Objective Structured Clinical Examination (OSCE) exam weeks).

SF leaders should also be aware that more staff may be needed to carry out new procedures adopted to contain the outbreak, such as cleaning and disinfecting simulation rooms and medical equipment after each training sessions. A definition of workflow and the resources needed to accomplish all the tasks is then recommended.

#### Materials and equipment

Over recent months, the world has witnessed a severe and mounting disruption to the global supply of PPE caused by rising demand [44]. Countries have been implementing extraordinary measures to ramp up production capacity, often by reorienting the manufacturers of nonmedical devices towards PPE production. However, as measures that have shattered economic activity across the countries are gradually lifted, there are already concerns about the availability of supplies that will be needed for workspaces. Therefore, procurement of the necessary equipment could become an increasing challenge for SF.

Another consideration would be to identify space to store a potentially large amount of PPE, as this should be clean, dry, easily accessible and protected from potentially damaging conditions.

#### Budget review

The challenge faced by SF is to fulfil the needs of educational programmes while respecting budgetary constraints [45]. Prices of PPE products have risen dramatically since the beginning of the COVID-19 outbreak: a sixfold increase for surgical masks and a doubling in the price of gowns [43]. SF managers should keep abreast of these new direct costs.

SF managers will face an increase in variable cost per hour due to the emerging expenses of new operational procedures, such as cleaning and disinfection and the large amount of PPE required.

The hours of room utilization for training activities will be reduced due to the time spent in non-training tasks, such as donning and doffing. In business terms, this could translate into decreasing billable hours taught per week and/or increase in cost per room utilization. SF managers should recognize how this wide range of measures could impact their economic sustainability, especially for those centres where charging end users is the preferred funding model.

In a recently published report, researchers laid out three scenarios for what the next 18 to 24 months might look like [46]. SF managers and executives should regularly revisit the steps outlined above and adjust measures accordingly. In addition, alternative solutions to continue educational activities in the event of reinstating lockdown restrictions, such as tele-simulation or virtual environments, might need to be incorporated as contingency plans.

### Focus point 10: Review of emergency plans and procedures

Finally, SF managers should consider how the reorganization of activities and potentially variable staff availability may influence the effectiveness of emergency management systems [47]. These systems should be updated, taking into account the new organizational layout. This may eventually lead to a thorough revision of policies and procedures.

## Conclusions

The speed of the COVID-19 pandemic is driving a profound transformation. The evolution of the crisis necessitates a review of all activities performed in SF to design our new normal.

We presented practical focus points and provided operational tips and mitigation recommendations to prepare the reader to reopen safely SF in the post-lockdown phase. The planning of future activities will have to be based not only on safety but also on flexibility principles.

We believe that sharing common methods consistent with national and international health guidelines, while taking into account the specific characteristics of the different contexts and centres, will ultimately foster dissemination of good practices.

Providing sage and evidence-based advices for specific aspects of reopening SF, we hope the manuscript *will* be informative for all policy-makers and stakeholders. It is also our hope that it will prompt research about the impact of such mitigation procedures and measures in different countries.

## Supplementary information

**Additional file 1.** COVID-19 “Health-Declaration” Form.

## Data Availability

Not applicable.

## Abbreviations

CDC: Centers for Disease Control and Prevention
EU: European Union
EU-OSHA: European Agency for Safety and Health at Work
NaClO: Sodium hypochlorite
OSCE: Objective Structured Clinical Examination
PPE: Personal protective equipment
SF: Simulation facilities
WHO: World Health Organization
