# Supplementary material for: COVID-19 crisis, safe reopening of simulation centres and the new normal: food for thought
Source: Adv Simul (Lond). 2020 Jul 16;5:13. doi: 10.1186/s41077-020-00131-3 (PMC7363498; doi:10.1186/s41077-020-00131-3)
Supplement: Supplementary file 1 — Additional file 1. COVID-19 “Health-Declaration” Form. [file 41077_2020_131_MOESM1_ESM.docx]

## Additional File 1. COVID-19 “Health-Declaration” Form

**LOGO OF CENTER**

**COVID-19 “Health-Declaration” Form**

The undersigned ........................................................................................................................................

born in ............................................................................ on (date of birth) ................................................... resident in ............................................... Province .......................................... ZIP code............................. in street/square .............................................................................................................................................

e-mail ......................................................................................................

phone number ……………………..……………………………………………………………………..

Training activity at the center ………………………………………………………… on (date) ………………………….

declares that the information below is true and accurate.

Have you had any of the following symptoms in the past 15 days?

| - Rise in body temperature above 37.5°C | YES | NO | N/A |
| --- | --- | --- | --- |
| - Sore throat | YES | NO | N/A |
| - Runny nose | YES | NO | N/A |
| - Cough | YES | NO | N/A |
| - Shortness of breath | YES | NO | N/A |
| - Vomiting | YES | NO | N/A |
| - Nausea | YES | NO | N/A |
| - Diarrhea | YES | NO | N/A |

DECLARATION OF CONSENT TO DATA PROCESSING PURSUANT TO ART. 23 OF LEGISLATIVE DECREE 196/2003.

The undersigned also declares to be informed, pursuant to and for the purposes of Art. 13 of Legislative Decree 196/2003, that the personal data collected will be processed, even by electronic means, exclusively within the procedure for which this declaration is made for and that, at any time, shall exercise the rights set forth by art. 7 of the Civil Code.

Date ________________ Signature _________________________________
